# Bacterial distribution and drug resistance patterns of urinary tract infection in tuberculosis patients: a 5-year retrospective study

**DOI:** 10.3389/fmicb.2026.1749129

**Published:** 2026-04-09

**Authors:** Jian Shui, Aichun Tan, Jianhua Pan, Guomin Shi

**Affiliations:** Department of Clinical Laboratory, The Affiliated Changsha Central Hospital, Hengyang Medical School, University of South China, Changsha, Hunan, China

**Keywords:** antimicrobial agents, bacterial distribution, drug resistance, tuberculosis, urinary tract infections

## Abstract

**Objective:**

To investigate the bacterial distribution and drug resistance profile of urinary tract infections (UTIs) in tuberculosis patients, and to provide evidence-based support for the rational clinical use of antimicrobial agents in this specific patient population.

**Methods:**

A retrospective analysis was performed on bacterial identification and antimicrobial susceptibility test results of tuberculosis patients complicated with UTIs in our hospital from January 2020 to December 2024. Data were processed via WHONET 5.6 and SPSS 21.0.

**Results:**

A total of 1,151 strains were isolated, including 878 Gram-negative strains (76.3%, mainly *Escherichia coli* 34.9% and *Klebsiella pneumoniae* 22.2%) and 273 Gram-positive strains (23.7%, predominantly *Enterococcus faecium* 15.6%). *Escherichia coli* showed low resistance to cefoperazone/sulbactam, amikacin, tigecycline and carbapenems (resistance rate < 5%). *Klebsiella pneumoniae* showed low resistance only to tigecycline (resistance rate 0%), while *Enterococcus faecium* showed low resistance to quinupristin/dalfopristin, linezolid, high concentrations of Streptomycin, high concentrations of Gentamicin, tigecycline and vancomycin (resistance rate < 5%). Additionally, *Klebsiella pneumoniae* resistance rates to multiple antimicrobials were significantly higher in male patients than in females (all *p* < 0.05). Severe tuberculosis patients had notably higher resistance rates of *Escherichia coli* to amoxicillin/clavulanate, ertapenem, and *Klebsiella pneumoniae* to multiple antimicrobials (all *p* < 0.05).

**Conclusion:**

Continuous monitoring of bacterial distribution and drug resistance in tuberculosis patients with UTIs is essential to improve diagnosis and treatment accuracy and guide rational clinical medication use.

## Introduction

Tuberculosis (TB), caused by *Mycobacterium tuberculosis*, remains a major global public health challenge, with China being one of the countries with the highest TB burden worldwide (Katherine et al., 2018; Chakaya et al., 2021). TB is a chronic infectious disease that often impairs immune function in patients, particularly in those with prolonged untreated courses, rendering them highly susceptible to secondary infections (Pai et al., 2011). Anti-tuberculosis medications, while essential for disease control, are also associated with multiple adverse reactions (e.g., hepatotoxicity, hematologic toxicity) that may further compromise host defense mechanisms, exacerbating the risk of secondary infections (Pai et al., 2011).

Urinary tract infections (UTIs) are among the most common secondary infections in clinical settings, ranking second only to respiratory infections, and are predominantly caused by Gram-negative bacteria (Dazhi et al., 2021; Gupta et al., 2017). UTIs not only increase the clinical management burden of primary diseases but also pose a significant threat to patient prognosis due to the global emergence of antibiotic resistance (Christaki et al., 2019; Sifri et al., 2019). Notably, the distribution of UTI pathogens and their antibiotic resistance patterns are highly region-specific and time-dependent, highlighting the necessity of localized epidemiological monitoring for rational antibiotic use (Gajdács et al., 2020). According to existing research, UTI drug resistance is closely associated with gender and disease severity, providing a basis for further exploration of resistant characteristics in specific populations. A study in Kosovo indicated that *Escherichia coli* and *Klebsiella pneumoniae* from male UTI patients showed higher resistance to most of the 13 tested antibiotics than those from females (Ilirjana et al., 2023), and Jie confirmed similar gender differences in male patients with urinary stones (Jie et al., 2021). Additionally, severe conditions correlate with elevated resistance—ICU patients exhibit higher resistance rates and more multidrug-resistant strains than general ward patients (Mihaela Elena and Carmen-Sarah, 2020; Yamei and Xinwen, 2022).

The coexistence of TB and UTI has attracted increasing clinical attention, yet relevant research remains scarce. Existing evidence has demonstrated that immunocompromised populations, including tuberculosis (TB) patients, are at a significantly elevated risk of urinary tract infections (UTIs) relative to the general population; such comorbidities may further result in delayed TB therapy, heightened treatment failure rates, and increased mortality (Kulchavenya and Cherednichenko, 2017; Manu et al., 2020; Niu T. et al., 2023; Niu X. et al., 2023). However, data on the pathogen spectrum and antibiotic resistance characteristics of UTIs in TB patients are still insufficient, which hinders the formulation of targeted prevention and treatment strategies.

To address this research gap, this study retrospectively analyzed the bacterial distribution and antimicrobial susceptibility profiles of UTIs in TB patients admitted to our hospital from 2020 to 2024. The objective was to provide evidence-based references for the clinical prevention and treatment of UTIs in TB patients and to promote the rational use of antibiotics.

## Materials and methods

### Strain source

This retrospective study was conducted from January 2020 to December 2024 at a tuberculosis-specialized hospital in Changsha, Hunan Province, central China. A total of 1,151 strains isolated from positive urine samples of tuberculosis patients were analyzed. The following exclusion criteria were strictly implemented: (1) Mixed bacterial infections (two or more distinct bacterial species meeting the positive culture criteria simultaneously); (2) Duplicate submitted samples from the same patient (only the culture results of the first submission were retained when the same strain was isolated consecutively from the same patient); (3) Samples with invalid collection or transportation (e.g., collection beyond the specified time window, contamination during transportation). This study received approval from the Ethics Committee of Changsha Central Hospital, and data from all patients were utilized exclusively for research purposes.

### Diagnostic criteria for urinary tract infection

UTI was diagnosed based on a combination of clinical symptoms and urine culture results. Clinical symptoms included frequent micturition, urgent micturition, dysuria, suprapubic pain, lumbago, fever (body temperature ≥ 38 °C), etc. Asymptomatic bacteriuria (ABU) was excluded from the study; specifically, individuals with positive urine culture findings but no concomitant clinical symptoms of UTI were not enrolled.

### Definition of severe tuberculosis

Severe tuberculosis was defined as tuberculosis patients who met the criteria for admission to the intensive care unit (ICU) due to tuberculosis-related complications, including severe respiratory failure requiring mechanical ventilation, septic shock caused by tuberculosis, multiple organ dysfunction syndrome secondary to tuberculosis, etc. Patients admitted to the ICU solely due to complicated urinary tract infections (e.g., urosepsis without tuberculosis progression) were not classified as severe tuberculosis cases.

### Culture and identification procedures

Urine samples were typically collected in the morning prior to the initiation of antibacterial treatment. Prior to collection, female patients cleaned their vulva, and male patients cleaned their urethral orifice. Clean midstream urine was then collected in sterile tubes and transported to the microbiology laboratory within 2 h for quantitative culture. The urine samples were inoculated onto 5% sheep blood agar (purchased from Zhengzhou Autobio Bioengineering Co. Ltd.) using sterile calibrated loops (10 μL). The inoculations were then incubated aerobically at 35 °C for 24–48 h, after which colony growth was observed.

A culture was considered positive for Gram-negative species if a single bacterial species reached a concentration of ≥10^5^ CFU/mL; similarly, growth of Gram-positive bacteria was defined as positive if it reached ≥10^4^ CFU/mL. Fungi and atypical pathogens (e.g., Mycoplasma, Chlamydia) were not included in this study, as the research focus was on bacterial urinary tract infections in tuberculosis patients, and the detection of fungi and atypical pathogens requires specialized culture media and detection methods, which were not within the scope of this study.

Bacterial identification was performed using either matrix-assisted laser desorption/ionization time-of-flight mass spectrometry (MALDI-TOF MS; Bruker, Germany) or the VITEK 2 Compact automated microbial identification system (bioMérieux, Craponne, France). For isolates identified via the VITEK 2 Compact system, Gram-positive (GP) cards (bioMérieux, Craponne, France) were utilized for Gram-positive bacteria, while Gram-negative (GN) cards (bioMérieux, Craponne, France) were used for Gram-negative bacteria.

### Drug sensitivity test and result interpretation

Subsequently, susceptibility tests were performed using the drug-sensitive reaction card (bioMérieux; Craponne, France) in conjunction with the Kirby-Bauer disk diffusion method. Quality control strains included *Escherichia coli* ATCC25922, *Staphylococcus aureus* ATCC25923, *Pseudomonas aeruginosa* ATCC27853, and *Klebsiella pneumoniae* ATCC700603.

Interpretation of results was based on the Clinical and Laboratory Standards Institute (CLSI) 2025 standard. For high-concentration standards of streptomycin and gentamicin, the criteria specified in CLSI 2025 were adopted: the high concentration of streptomycin was defined as 1,000 μg/mL, and the high concentration of gentamicin was defined as 500 μg/mL, which were used for detecting high-level resistance of bacteria to these two aminoglycoside antibiotics. For drug resistance rate calculation, the following processing method was adopted: only strains identified as “resistant” were counted in the resistant group; strains with “intermediate” susceptibility were excluded from both resistant and sensitive groups and not included in the resistance rate calculation. This is because many antibiotics are concentrated in urine and can effectively eradicate certain bacteria in the urinary tract despite intermediate susceptibility, thus avoiding overestimation of drug resistance rates.

### Statistical analysis

The susceptibility test results were processed using WHONET 5.6 software (WHO, Geneva, Switzerland). The culture results and clinical data were analyzed using SPSS 21.0 software (Chicago, IL, United States). Categorical variables were expressed as counts and percentages, and comparisons between groups were conducted using Pearson’s chi-square test; Fisher’s exact test was used when the expected frequency of any cell in the contingency table was <5. For the analysis of time trends of drug resistance rates, the trend chi-square test was used to evaluate the changes in resistance rates over the study period (2020—2024). A two-tailed *p* value < 0.05 was considered statistically significant.

## Results

A total of 1,151 non-repetitive bacterial strains were isolated from urine samples of tuberculosis patients, among which 878 strains (76.3%) were Gram-negative bacteria and 273 strains (23.7%) were Gram-positive bacteria (Table 1). In terms of prevalence, the top three pathogens were *Escherichia coli*, *Klebsiella pneumoniae*, and *Enterococcus faecium* (Table 1).

**Table 1:** Distribution and composition of 1,151 strains of pathogenic bacterial strains.

Pathogen	Strain (*n*)	Percentage (%)
Gram negative	878	76.3
*Escherichia coli*	401	34.9
*Klebsiella pneumoniae*	256	22.2
*Proteus mirabilis*	49	4.3
*Enterobacter cloacae*	37	3.2
*Acinetobacter baumannii*	32	2.8
*Pseudomonas aeruginosa*	13	1.1
Others	90	7.8
Gram positive	273	23.7
*Enterococcus faecium*	179	15.6
*Enterococcus faecalis*	34	3.0
*Staphylococcus haemolyticus*	27	2.3
*Staphylococcus epidermidis*	15	1.3
*Streptococcus agalactiae*	5	0.4
*Staphylococcus aureus*	4	0.3
Others	9	0.8
Total	1,151	100

*Escherichia coli* exhibited low resistance to cefoperazone/sulbactam, amikacin, tigecycline, imipenem, meropenem, and ertapenem, with the pooled resistance rate (across all study years) consistently below 5%. Notably, no tigecycline- or meropenem-resistant *Escherichia coli* isolates were detected (Table 2). In contrast, *Klebsiella pneumoniae* presented varying levels of resistance to commonly used antibiotics, and its pooled resistance rate (across all study years) to piperacillin/tazobactam, ceftriaxone, cefuroxime, levofloxacin, and trimethoprim/sulfamethoxazole all exceeded 50%. Nevertheless, no tigecycline-resistant *Klebsiella pneumoniae* strains were identified (Table 3). Additionally, *Enterococcus faecium* isolates derived from urinary tract infections (UTIs) in tuberculosis patients showed no resistance to quinupristin/dalfopristin, linezolid, high-concentration streptomycin, high-concentration gentamicin, or tigecycline. Although the majority of *Enterococcus faecium* strains maintained low resistance to vancomycin, a small proportion of vancomycin-resistant isolates has emerged in this cohort (Table 4).

**Table 2:** Antibiotic resistance rate of *Escherichia coli* to antibiotics.

Antibiotics	Resistance rate (%)					
	Pooled (*n* = 401)	2020 (*n* = 105)	2021 (*n* = 80)	2022 (*n* = 69)	2023 (*n* = 66)	2024 (*n* = 81)
Piperacillin/tazobactam	13.6	11.7	15.8	17.9	9.1	13.9
Amoxicillin/clavulanate	18.4	12.8	19.0	25.0	20.4	18.3
Cefoperazone/sulbactam	4.0	4.8	5.1	4.3	1.5	3.7
Ceftriaxone	60.4	59.0	64.1	59.1	59.2	60.3
Ceftazidime	32.0	32.4	35.0	37.7	26.2	28.4
Cefuroxime	63.7	62.8	73.4	63.6	55.1	61.7
Cefoxitin	20.7	20.5	23.4	25.0	16.3	18.6
Cefepime	12.1	8.8	18.2	16.4	7.7	10.1
Amikacin	2.8	3.8	1.2	1.4	1.5	5.9
Levofloxacin	74.1	68.6	72.5	79.7	71.2	80.2
Tigecycline	0.0	0.0	0.0	0.0	0.0	0.0
Imipenem	0.5	0.0	0.0	0.0	0.0	2.5
Meropenem	0.0	0.0	0.0	0.0	0.0	0.0
Ertapenem	1.0	1.3	0.0	0.0	0.0	2.9
Trimethoprim/sulfamethoxazole	57.4	63.8	58.8	47.8	59.1	54.3

**Table 3:** Antibiotic resistance rate of *Klebsiella pneumoniae* to antibiotics.

Antibiotics	Resistance rate (%)					
	Pooled (*n* = 256)	2020 (*n* = 46)	2021 (*n* = 51)	2022 (*n* = 48)	2023 (*n* = 43)	2024 (*n* = 68)
Piperacillin/tazobactam	53.9	47.8	43.1	50.0	51.2	66.7
Amoxicillin/clavulanate	38.6	28.9	25.8	30.8	50.0	52.5
Cefoperazone/sulbactam	28.9	19.6	23.5	25.0	37.2	36.2
Ceftriaxone	59.2	57.9	61.3	65.4	68.6	47.1
Ceftazidime	48.4	34.8	49.0	52.1	51.2	52.2
Cefuroxime	65.2	57.9	61.3	69.2	70.7	66.7
Cefoxitin	39.1	31.6	36.7	40.0	38.6	46.2
Cefepime	50.0	39.1	47.1	45.8	55.8	58.0
Amikacin	24.1	23.9	19.6	29.2	25.6	20.7
Levofloxacin	72.4	68.9	68.6	72.3	72.1	78.3
Tigecycline	0.0	0.0	0.0	0.0	0.0	0.0
Imipenem	21.2	15.2	19.6	25.0	19.0	24.6
Meropenem	23.3	25.0	20.0	22.7	24.1	24.0
Ertapenem	20.9	15.8	19.4	24.0	17.3	25.0
Trimethoprim/sulfamethoxazole	72.5	77.8	60.8	68.8	76.7	78.3

**Table 4:** Antibiotic resistance rate of *Enterococcus faecium* to antibiotics.

Antibiotics	Resistance rate (%)					
	Pooled (*n* = 179)	2020 (*n* = 46)	2021 (*n* = 30)	2022 (*n* = 43)	2023 (*n* = 31)	2024 (*n* = 29)
Levofloxacin	97.8	93.5	100	97.7	100	100
Ciprofloxacin	97.8	93.5	100	97.7	100	100
Penicillin G	98.9	95.7	100	100	100	100
Ampicillin	97.8	93.5	96.7	100	100	100
Nitrofurantoin	80.4	80.4	83.3	76.7	74.2	90.0
Erythromycin	84.2	80.4	89.2	86.0	83.9	83.3
Quinupristin/dalfopristin	0.0	0.0	0.0	0.0	0.0	0.0
Linezolid	0.0	0.0	0.0	0.0	0.0	0.0
High concentration of streptomycin	0.0	0.0	0.0	0.0	0.0	0.0
High concentration of gentamicin	0.0	0.0	0.0	0.0	0.0	0.0
Tetracycline	53.1	56.5	60.0	48.8	48.4	53.3
Tigecycline	0.0	0.0	0.0	0.0	0.0	0.0
Vancomycin	3.4	0.0	0.0	0.0	9.7	10.3

The Cochran-Armitage trend chi-square test was employed to analyze the linear trends of antimicrobial resistance rates of the three dominant pathogens over the 5-year period. The results revealed distinct trend patterns among different pathogens and antibiotics. For *Escherichia coli*, no statistically significant temporal trends were observed in resistance rates to most antibiotics. Specifically, the resistance rate to imipenem exhibited a statistically significant increasing trend (trend χ^2^ = 4.239, *p* = 0.040). Resistance rates to the remaining antibiotics remained stable throughout the study period (all *p* > 0.05). In contrast, *Klebsiella pneumoniae* exhibited statistically significant increasing trends in resistance to piperacillin/tazobactam (trend χ^2^ = 5.352, *p* = 0.021), amoxicillin/clavulanate (trend χ^2^ = 12.798, *p* < 0.001), cefoperazone/sulbactam (trend χ^2^ = 5.826, *p* = 0.016), and cefepime (trend χ^2^ = 4.261, *p* = 0.039). Resistance rates to the remaining antibiotics did not show significant linear temporal trends (all *p* > 0.05). For *Enterococcus faecium*, a significant increasing trend in Vancomycin resistance was detected (trend χ^2^ = 8.833, *p* = 0.003), with resistance rates rising from 0% in 2020–2022 to 9.7% in 2023 and 10.3% in 2024. No significant temporal trends were observed for other antibiotics, including Levofloxacin, Penicillin G, and Tetracycline (all *p* > 0.05).

Among the 401 *Escherichia coli* strains isolated, 120 were derived from urine samples of male patients and 281 from female counterparts. For the 256 *Klebsiella pneumoniae* strains, 129 were obtained from male patients’ urine specimens, with the remaining 127 isolated from those of female patients. As for the 179 *Enterococcus faecium* strains, 93 were recovered from male patients’ urine samples, whereas 86 were from female patients.

Notably, *Klebsiella pneumoniae* strains isolated from tuberculosis patients with urinary tract infection (UTI) exhibited gender-associated differences in drug resistance, whereas no statistically significant differences were observed in the resistance profiles of *Escherichia coli* (Table 5) and *Enterococcus faecium* (Table 6) between the two genders. Specifically, in male tuberculosis patients, the resistance rates of *Klebsiella pneumoniae* to piperacillin/tazobactam, amoxicillin/clavulanate, cefoperazone/sulbactam, ceftriaxone, ceftazidime, cefuroxime, cefoxitin, cefepime, amikacin, levofloxacin, imipenem, meropenem, and trimethoprim/sulfamethoxazole were significantly higher than those detected in female patients (*p* < 0.05) (Table 5).

**Table 5:** Antibiotic resistance in key gram-negative bacteria (*E. coli*, *K. pneumoniae*) by patient gender.

Antibiotics	*Escherichia coli*			*Klebsiella pneumoniae*		
	Male	Female	*P*	Male	Female	*P*
Piperacillin/tazobactam	13.3	13.7	0.959	63.0	44.6	0.004*
Amoxicillin/clavulanate	14.5	19.9	0.171	46.3	31.5	0.014*
Cefoperazone/sulbactam	6.7	2.9	0.131	38.0	19.7	0.001*
Ceftriaxone	67.5	57.7	0.065	68.1	50.7	0.004*
Ceftazidime	35.3	30.6	0.387	56.6	39.4	0.006*
Cefuroxime	69.9	61.3	0.093	74.6	56.3	0.003*
Cefoxitin	26.5	18.5	0.066	46.5	31.9	0.020*
Cefepime	13.4	11.4	0.583	56.9	42.5	0.024*
Amikacin	0.8	3.7	0.232	31.7	15.6	0.003*
Levofloxacin	77.5	72.6	0.305	78.9	65.9	0.020*
Tigecycline	0.0	0.0	–	0	0	–
Imipenem	0.0	0.7	1.000	27.9	14.3	0.007*
Meropenem	0.0	0.0	–	29.9	16.1	0.006*
Ertapenem	0.0	1.4	0.322	25.0	16.5	0.102
Trimethoprim/sulfamethoxazole	61.7	55.5	0.254	82.2	62.7	0.001*

*Statistically significant based on chi-square test (*p* < 0.05). Where a chi-square test was not suitable, Fisher’s exact probability test was used.

**Table 6:** Antibiotic resistance of *Enterococcus faecium* by patient gender.

Antibiotics	*Enterococcus faecium*		
	Male	Female	*P*
Levofloxacin	97.8	97.7	1.000
Ciprofloxacin	97.8	97.7	1.000
Penicillin G	98.9	98.8	1.000
Ampicillin	97.8	97.7	1.000
Nitrofurantoin	77.9	81.7	0.557
Erythromycin	82.8	85.6	0.550
Quinupristin/dalfopristin	0.0	0.0	–
Linezolid	0.0	0.0	–
High concentration of streptomycin	0.0	0.0	–
High concentration of gentamicin	0.0	0.0	–
Tetracycline	52.7	53.5	0.915
Tigecycline	0.0	0.0	–
Vancomycin	4.3	2.4	0.750

Of the 401 *Escherichia coli* strains isolated, 28 were recovered from urine samples of intensive care unit (ICU) patients, with the remaining 373 derived from those of general ward patients. For the 256 *Klebsiella pneumoniae* strains, 42 were obtained from ICU patients’ urine specimens, whereas 214 were isolated from general ward patients. With respect to the 179 *Enterococcus faecium* isolates, 59 were retrieved from ICU patients’ urine samples, and 120 from general ward counterparts.

Notably, *Escherichia coli* and *Klebsiella pneumoniae* strains isolated from tuberculosis patients complicated with urinary tract infection exhibited differential resistance patterns to commonly used antibiotics based on disease severity (Table 7), whereas no statistically significant differences were observed in the resistance profiles of *Enterococcus faecium* isolates between the two groups (Table 8). Specifically, the resistance rates of *Escherichia coli* from urine samples of ICU-admitted tuberculosis patients with urinary tract infection to amoxicillin/clavulanate and ertapenem were significantly higher than those from their counterparts in general wards (*p* < 0.05) (Table 7). In contrast, the resistance rates of *Klebsiella pneumoniae* isolates from ICU tuberculosis patients with urinary tract infection to piperacillin/tazobactam, amoxicillin/clavulanate, cefoperazone/sulbactam, ceftriaxone, ceftazidime, cefuroxime, cefoxitin, cefepime, imipenem, meropenem, and ertapenem were markedly elevated compared with those from general ward tuberculosis patients (*p* < 0.05) (Table 7).

**Table 7:** Antibiotic resistance of key gram-negative bacterial isolates (*E. coli*, *K. pneumoniae*) in ICU and general ward patients.

Antibiotics	*Escherichia coli*			*Klebsiella pneumoniae*		
	ICU	General	*P*	ICU	General	*P*
Piperacillin/tazobactam	25.0	12.6	0.117	73.3	50.1	0.005^*^
Amoxicillin/clavulanate	39.8	16.9	0.003^*^	75.9	31.4	0.000^*^
Cefoperazone/sulbactam	10.7	3.5	0.166	53.8	23.8	0.000^*^
Ceftriaxone	60.0	60.6	1.000	88.5	53.2	0.000^*^
Ceftazidime	35.7	31.8	0.677	63.1	45.5	0.025^*^
Cefuroxime	66.7	63.7	0.667	94.5	59.5	0.000^*^
Cefoxitin	26.7	20.4	0.560	67.9	33.6	0.000^*^
Cefepime	21.4	11.5	0.214	70.5	45.9	0.002^*^
Amikacin	3.2	2.8	0.554	34.5	22.1	0.114
Levofloxacin	71.4	74.4	0.717	82.3	70.5	0.090
Tigecycline	0.0	0.0	–	0	0	–
Imipenem	3.6	0.3	0.135	44.2	16.8	0.000^*^
Meropenem	0.0	0.0	–	35.9	21.0	0.040^*^
Ertapenem	6.7	0.7	0.041^*^	57.0	13.9	0.000^*^
Trimethoprim/sulfamethoxazole	64.3	56.8	0.442	70.4	72.9	0.845

*Statistically significant based on chi-square test (*p* < 0.05). Where a chi-square test was not suitable, Fisher’s exact probability test was used.

**Table 8:** Antibiotic resistance of *Enterococcus faecium* in ICU and general ward patients.

Antibiotics	*Enterococcus faecium*		
	ICU	General	*P*
Levofloxacin	100	96.7	0.379
Ciprofloxacin	100	96.7	0.379
Penicillin G	100	98.4	1.000
Ampicillin	100	96.7	0.379
Nitrofurantoin	78.0	82.0	0.557
Erythromycin	86.1	83.2	0.591
Quinupristin/dalfopristin	0.0	0.0	–
Linezolid	0.0	0.0	–
High concentration of streptomycin	0.0	0.0	–
High concentration of gentamicin	0.0	0.0	–
Tetracycline	54.2	53.3	0.909
Tigecycline	0.0	0.0	–
Vancomycin	3.4	3.3	1.000

## Discussion

Secondary urinary tract infections (UTIs) in tuberculosis (TB) patients are caused by pathogens other than *Mycobacterium tuberculosis*, with their occurrence closely linked to immune dysfunction, complex comorbidities, invasive procedures, and irrational antibiotic use. These infections add a substantial burden to the clinical management of TB patients and may even compromise their prognosis. Clarifying the pathogen distribution and antibiotic resistance profiles in TB patients complicated with UTIs is therefore pivotal for guiding rational clinical medication and curbing the spread of drug-resistant strains. In this study, we analyzed pathogens and their antibiotic resistance patterns in urine samples from TB patients with concurrent UTIs, and by integrating targeted comparisons with existing literature (Niu T. et al., 2023; Niu X. et al., 2023; Ana et al., 2015; Qiwen et al., 2017), we further interpreted the clinical and scientific implications of our findings, clarified the study limitations, and proposed directions for future research.

Gram-negative bacilli were the predominant pathogens in TB patients with UTIs, consistent with the findings of Kulchavenya (Kulchavenya and Cherednichenko, 2017), who also focused on this specific population (TB patients with UTIs) and reported the highest proportion of Gram-negative bacilli in the pathogen spectrum. This consistency underscores the shared characteristics of UTI pathogen profiles among TB patients. As the dominant pathogen, *Escherichia coli* exhibited a high detection rate, which not only aligns with the general UTI pathogen distribution but also corroborates the results of Ana and Anja—who studied infectious disease patients with UTIs (Ana et al., 2015; Anja et al., 2024)—thus highlighting *Escherichia coli*’s universal pathogenic dominance across diverse UTI-affected populations. Notably, *Klebsiella pneumoniae* ranked second in detection rate, while *Enterococcus faecium* accounted for 15.6% of isolates—marking a striking difference from Carmen’s study on uncomplicated UTI patients (non-TB population), where *Enterococcus* spp. only constituted 3.5%(Carmen et al., 2017). This discrepancy stems primarily from the uniqueness of our study population: most samples were collected from hospitalized TB patients, who are predominantly elderly with impaired immune function, and some have indwelling catheters. Indwelling catheters and immunosuppression are well-established high-risk factors for Gram-positive bacterial UTIs, a finding consistent with Lok Bahadur’s research on critically ill patients with UTIs (Lok Bahadur et al., 2019), thus confirming the specificity of pathogen spectra in special populations like hospitalized TB patients.

Antibiotic resistance analysis revealed significant resistance of *Escherichia coli* to commonly used cephalosporins, a phenomenon that must be contextualized within TB clinical practice. Clinically, cephalosporins—particularly third-generation agents—are frequently used empirically for concurrent infections in TB patients due to their broad antibacterial spectrum, and long-term irrational use exerts selective pressure on pathogens, driving the emergence and dissemination of drug-resistant strains. Consistent with Mei’s study focusing on urinary calculi patients with concurrent urinary tract infections (Mei et al., 2024), our study further confirmed that cefoperazone/sulbactam, amikacin, tigecycline, and carbapenems retain robust antibacterial activity against *Escherichia coli*. This inter-study consistency not only validates the cross-population efficacy of these agents against uropathogenic *Escherichia coli* but also furnishes evidence-based guidance for the rational selection of antimicrobials in TB patients with concurrent urinary tract infections. The resistance rate of *Escherichia coli* to levofloxacin exceeded 70%, which is highly consistent with Qiwen’s report of an approximate 30% levofloxacin susceptibility rate in hospitalized UTI patients (Qiwen et al., 2017). This consistency confirms that quinolones have significantly diminished clinical efficacy in TB patients with UTIs. While some studies suggest optimizing dosage regimens and enhancing renal toxicity monitoring may improve levofloxacin’s efficacy, the high resistance rate observed herein—coupled with quinolones’ potential side effects—mandates caution when using these agents empirically for this specific cohort.

*Klebsiella pneumoniae* showed low resistance to tigecycline but exhibited resistance rates exceeding 45% to most commonly used antibiotics, with carbapenem resistance reaching over 20%. This trend aligns with Caroline’s research on multidrug-resistant *Klebsiella pneumoniae* infections, highlighting the risk of multidrug-resistant strain transmission in TB patients—a concern requiring heightened clinical vigilance (Caroline et al., 2021). *Enterococcus faecium* displayed low vancomycin resistance; however, an upward trend in vancomycin resistance was observed in the latter phase of the study, consistent with Wei’s conclusions on hospital-acquired *Enterococcus* infections (Wei et al., 2018). This emphasizes the need for intensified resistance monitoring of such strains in TB patient populations.

This study further explored gender-based differences in the antibiotic resistance of strains isolated from UTIs in TB patients. The results showed that the resistance rates of *Klebsiella pneumoniae* isolated from male TB patients with concurrent UTIs to multiple clinically commonly used antibiotics were significantly higher than those from female patients. The discovery of this gender-related resistance difference is consistent with the conclusions of existing studies. A study conducted by Ilirjana in Kosovo indicated that *Escherichia coli* and *Klebsiella pneumoniae* isolated from the urine of male UTI patients exhibited higher resistance to most of the 13 antibiotics involved in the study compared to female patients (Ilirjana et al., 2023). Similarly, a study by Jie found that *Escherichia coli* and *Klebsiella pneumoniae* isolated from urine samples of male patients with urinary calculi complicated by UTIs showed significantly stronger resistance to commonly used antibiotics than those from female patients (Jie et al., 2021). These studies corroborate the findings of the present study, suggesting that the pathogen spectrum and antibiotic resistance patterns of UTIs may have gender-specific differences, and this characteristic may exist in UTI populations with different underlying disease backgrounds.

Integrating the clinical characteristics and physiological differences of TB patients, this study proposes three potential mechanistic hypotheses for the aforementioned gender-based resistance differences. These hypotheses are grounded in existing medical literature and are proposed to guide future mechanistic research: First, anatomical differences in the urinary tract may play a role. The longer and curved structure of the male urethra has been documented to facilitate latent colonization of uropathogens following infection. This anatomical feature could potentially hinder the complete elimination of bacteria by antibiotics, and as chronic infection states are known to induce the production and proliferation of drug-resistant strains. Recent research on bacterial persistence has demonstrated that physiologically dormant persister cells can tolerate high levels of antibiotics and may give rise to resistant offspring, particularly in chronic infections (Skow et al., 2020). Second, behavioral and lifestyle factors may contribute to the disparity. It is well-established that male TB patients have a relatively higher prevalence of unhealthy lifestyles such as smoking and heavy alcohol consumption compared to females. Both smoking and alcohol abuse are known to impair pulmonary and systemic immune function, reducing the host’s ability to clear pathogens and thereby potentially increasing the risk of multidrug-resistant infections (Kadam et al., 2019; Song et al., 2022). Third, the higher incidence of specific comorbidities in males could be a contributing factor. Males are more prone to urological conditions such as prostate diseases and urinary obstruction (Akinpelu et al., 2024; Iwodi et al., 2024). These comorbidities can prolong the infection course and necessitate extended or repeated courses of antibiotic therapy, which elevates resistance rates by intensifying the selective pressure of antibacterial drugs. Against this backdrop, conducting specialized surveys on the pathogen spectrum and antibiotic resistance patterns of UTIs in TB patients of different genders carries notable clinical implications and academic merit. Clarifying the gender differences in resistance and their potential mechanisms in this population can provide an evidence-based basis for formulating individualized empirical anti-infective treatment regimens in clinical practice. This approach would help optimize medication strategies, reduce the irrational use of antibacterial drugs, and thereby curb the emergence and spread of drug-resistant strains from the source. It is important to note that the above hypotheses are speculative and require further verification and refinement through multivariate analysis incorporating confounding factors such as comorbidities and lifestyles, combined with prospective study designs.

The tuberculosis intensive care unit (TB ICU) serves as a critical rescue facility for severe cases within designated tuberculosis hospitals, admitting patients characterized by severe conditions, complex underlying diseases, compromised immune systems, and prolonged hospitalizations. Life-saving invasive interventions, such as endotracheal intubation and deep vein catheterization, are frequently required in this cohort; coupled with the extensive administration of broad-spectrum antibiotics for anti-infective prophylaxis and treatment post-admission, these factors collectively elevate the risk of secondary bacterial infections and impose strong selective pressure on pathogens. Consistent with this clinical context, our study demonstrated that the antibiotic resistance rates of *Escherichia coli* isolated from patients with severe tuberculosis to amoxicillin/clavulanate and ertapenem were significantly higher than those observed in non-severe tuberculosis patients. More strikingly, *Klebsiella pneumoniae* isolates from severe cases exhibited elevated resistance across an extensive panel of antimicrobials—including piperacillin/tazobactam, amoxicillin/clavulanate, cefoperazone/sulbactam, ceftriaxone, ceftazidime, cefuroxime, cefoxitin, cefepime, imipenem, meropenem, and ertapenem—when compared with the non-severe group.

The observed disparity in antibiotic resistance rates between critically ill and non-critically ill TB patients essentially reflects a direct correlation between treatment intensity and the emergence of drug resistance, a finding corroborated by numerous prior studies (Eilish et al., 2018; Mihaela Elena and Carmen-Sarah, 2020; Yamei and Xinwen, 2022). These investigations have consistently confirmed that the intensive use of broad-spectrum, high-potency antibiotics in ICU settings drives the development of significant antibiotic resistance in bacteria isolated from critically ill patients. In TB ICUs specifically, the confluence of severe immune dysfunction, invasive procedures, and early empiric broad-spectrum antibiotic exposure exerts robust selective pressure on pathogens, which in turn significantly increases the detection rate of drug-resistant strains.

This evidence underscores the critical importance of optimized antibiotic stewardship in TB ICUs and provides a key evidence-based basis for clinical practice. Pending the results of drug susceptibility testing (DST), empirical antimicrobial regimens must be tailored to the local or institutional pathogen distribution and resistance patterns to avoid the indiscriminate use of broad-spectrum antibiotics that fuels resistance development. Once DST results are available, prompt de-escalation to targeted therapy is essential to reduce unnecessary antibiotic exposure and curb the proliferation and transmission of multidrug-resistant (MDR) or extensively drug-resistant (XDR) strains.

Building on our findings and integrating clinical implications, we propose the following tailored recommendations for the empirical treatment of urinary tract infections (UTIs) in TB patients: First, for non-critically ill patients, empirical therapy should prioritize cefoperazone/sulbactam and amikacin. This recommendation is supported by evidence showing high susceptibility of amikacin (up to 96%) against uropathogens, including ESBL-producing strains, while resistance to third-generation cephalosporins and levofloxacin is notably high (Ishii et al., 2021). Third-generation cephalosporins and levofloxacin should therefore be avoided as first-line agents to mitigate the risk of selecting for resistant strains. Second, for male TB patients with UTIs, clinicians must remain vigilant for multidrug-resistant (MDR) infections. Given that MDR Enterobacteriaceae are a growing concern and are associated with worse clinical outcomes (Tamma et al., 2012), we recommend appropriately enhancing the intensity of empirical antibacterial therapy and expediting drug susceptibility testing (DST) to guide targeted adjustments. Third, for UTI patients admitted to TB ICUs, initial empirical treatment should employ a carbapenem-based combination regimen. Evidence suggests that for severe infections caused by MDR pathogens, carbapenems are the drugs of choice (Perez et al., 2016; Doi, 2019). Including tigecycline in the combination ensures coverage of carbapenem-resistant Gram-negative and MDR Gram-positive pathogens, with subsequent de-escalation based on DST confirmation. Fourth, beyond antimicrobial stewardship, clinical practice should emphasize the reduction of infection-related risk factors. The duration of invasive procedures (e.g., indwelling urinary catheters) must be strictly limited. Implementing enhanced nursing interventions and nurse-driven protocols is critical to reduce the incidence of catheter-associated UTIs (CAUTIs) (Lo et al., 2014), thereby minimizing the need for antibiotic use and controlling resistance at its source.

Several limitations of this study should be acknowledged. First, as a single-center study conducted at a specialized tuberculosis hospital, the findings may have limited generalizability to other institutions or patient populations with different clinical characteristics. Second, given its retrospective design, the potential for incomplete or missing data cannot be overlooked. Third, this study did not include a molecular investigation into the underlying mechanisms of drug resistance. Future prospective, multicenter studies that integrate analyses of molecular resistance mechanisms are needed to validate and extend our conclusions, ultimately offering a more comprehensive evidence base for the optimized management of TB patients with UTIs.

In conclusion, this study clarifies the pathogen distribution and antibiotic resistance characteristics of UTIs in TB patients. These findings provide valuable insights into empirical antibiotic therapy, complementing those of existing studies. Our results underscore the importance of strengthening antibiotic stewardship in TB patients—particularly those in ICUs and male patients—to curb the spread of multidrug-resistant strains.

## Data Availability

The original contributions presented in the study are included in the article/supplementary material, further inquiries can be directed to the corresponding authors.
